# Larval ecology of *Anophelescoluzzii* in Cape Coast, Ghana: water quality, nature of habitat and implication for larval control

**DOI:** 10.1186/s12936-015-0989-4

**Published:** 2015-11-11

**Authors:** Andreas A. Kudom

**Affiliations:** Department of Entomology and Wildlife, School of Biological Sciences, University of Cape Coast, Cape Coast, Ghana

**Keywords:** *Anopheles*, Habitat ecology, *Culex*, Larval control, Pollution, Urbanization

## Abstract

**Background:**

There is a growing interest in larval control intervention to supplement existing malaria control strategies, particularly in urban areas. However, effective implementation requires a good understanding of habitat ecology of *Anopheles* mosquitoes. Clean water bodies have long been reported by several studies as a preferred breeding habitat for *Anopheles gambiae*. Other studies have also reported the breeding of *An. gambiae* in polluted water bodies. However, the term clean or polluted is mostly based on visual examination and is not well defined. This study was conducted with the aim of assessing water quality in *Anopheles* breeding habitats and the practicability of larval control in Cape Coast, Ghana.

**Methods:**

A larval survey was conducted for 15 months in Cape Coast. In individual breeding habitats, habitat characteristics, physicochemical parameters and bacterial fauna were measured in both *Anopheles* positive breeding (APL) habitats and habitats colonized by only *Culex* species. The sibling species of *An. gambiae* were identified using PCR assay.

**Results:**

*Anopheles coluzzii* dominated in almost all the APL habitats found in this study. The habitats had high levels of salinity and ammonium ions. However, ammonium ions were significantly higher (p = 0.001) in habitats colonized by only *Culex* larvae compared to APL habitats. About 47 % of the habitats that were colonized by only *Culex* larvae had no measurable dissolved oxygen while *An. coluzzii* was absent in such habitats. High concentration of faecal bacteria confirmed faecal contamination in both groups of breeding habitats.

**Conclusions:**

From the results, it was evident that larval stages of *An. coluzzii* have tolerance to high levels of salinity and organic pollution in breeding habitats. However, its level of tolerance to organic pollution is probably lower than *Culex* larvae. The nature of breeding habitats found in the city demonstrates the opportunistic behaviour of *An. coluzzii* and how its breeding requirements are so intimately intertwined with the haphazard and uncontrolled human activities in the urban area. Considering the nature of APL habitats, larval control intervention could greatly reduce *Anopheles* population. However, improving basic hygiene and sanitation in the city could even make larval control intervention more practical and cost effective.

## Background

Most African cities are undergoing rapid urbanization. Presently, about 53 % of Ghana’s entire population live in urban areas and this is projected to increase up to 70 % by 2050 [1]. This has rejuvenated interest in the challenges of urban malaria control in the country. Current malaria vector control measures include use of long lasting insecticidal nets and indoor residual spraying. However, these measures alone may be insufficient to achieve malaria elimination in Ghana and in many other endemic countries. A major limitation of the current control measures is the inability to cover the full spectrum of locations where mosquito exposure occurs. The existence of outdoor biting mosquitoes could prevent attainment of malaria elimination, even if it involves a small percentage of malaria vectors [2]. Additional malaria control measures are clearly required. Over the past decade there has been growing interest in larval control intervention to supplement existing malaria control strategies [3–5]. However, effective implementation requires a good understanding of habitat ecology of *Anopheles* mosquitoes, which is limited, especially in many urban areas in Ghana.

Comprehensive data on habitat ecology of *Anopheles gambiae* in urban areas in Ghana dates back more than a century [6]. Since then, there have been few studies on habitat ecology of *Anopheles* species, which in most cases targets specific ecological or land use settings, such as urban agricultural areas [7, 8]. Indeed, urban agriculture is currently cited as a major breeding site for *An. gambiae* in many urban areas. In Kumasi (Ghana’s second largest city), for example, urban agriculture is reported to be responsible for the production of over 80 % of malaria mosquitoes in the city [7]. Breeding does take place in some urban areas without any important urban agriculture; however, information on *Anopheles* larval habitats is woefully inadequate in such urban areas. Clear ecological characteristics of *Anopheles* larval breeding requirements have not been identified for urban settings. Small, temporary sunny water bodies, relatively clean and mostly without overhanging vegetation have been recognized by several studies as preferred breeding habitats for *An. gambiae* [9]. Other studies have also reported the breeding of *An. gambiae* in large permanent and polluted water bodies [10, 11]. However, the term clean or polluted habitat is mostly based on visual examination and is not well defined. Quantifying water quality or pollution in *Anopheles* breeding habitats may give more insight into *Anopheles* breeding requirements, particularly in urban settings.

The present study was conducted in Cape Coast to gain more insight into *Anopheles* larval ecology in urban settings and assess the practicability of larval control intervention in the city. Cape Coast is an urban area in the coastal savannah region of Ghana with a rich mosaic of wetland habitats but without any major commercial vegetable farming. Human activities (partly due to rapid urbanization) and/or climatic change has created or modified various larval habitats in the metropolis. Similar to the rest of the country, malaria transmission occurs throughout the year. However, Cape Coast is one of the two metropolises with high percentage malaria parasitaemia in children and the lowest percentage of households that own insecticide-treated nets among the large urban areas in Ghana (Multiple Indicator Cluster Survey 2011, Ghana Statistical Service). There is limited information on malaria vector composition and their habitat ecology in the city.

The main objective of the study was to determine *Anopheles* species composition in the city and characterize their breeding habitats, with particular emphasis on water quality in the breeding habitats. Since *Culex* larvae are known to colonize polluted habitats, water quality of the *Anopheles* breeding habitats was compared to that of habitats that were colonized by *Culex* larvae only. A larval survey was conducted for 15 months in different landscape and land use settings in Cape Coast. *Anopheles coluzzii* was found to be the dominant *Anopheles* species, breeding in organic polluted habitats. However, the level of organic pollution appears to be lower in *An. coluzzii* breeding habitats compared to habitats that were colonized by only *Culex* larvae. Almost all the habitats were anthropogenic in nature and it seems an improvement of basic hygiene and sanitation with regards to waste management in the city could prevent the formation of many of the *Anopheles* breeding habitats in Cape Coast.

## Methods

### Study sites

**Fig. 1:**
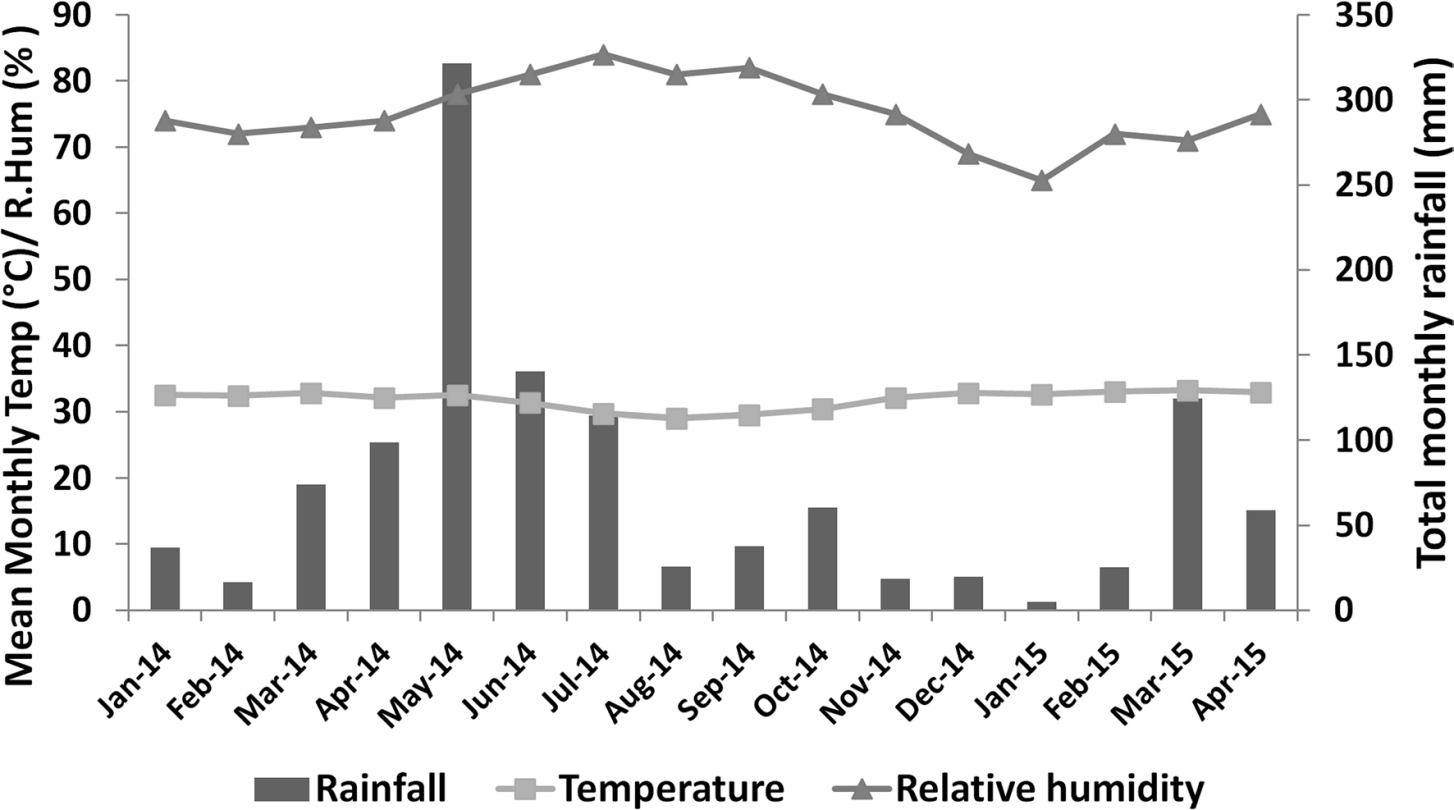
Mean monthly temperature, relative humidity and total monthly rainfall in Cape Coast from January 2014 to April 2015. *Temperature* mean maximum daily temperature; *relative humidity* mean daily relative humidity at 12 noon

Cape Coast is about 165 km west of Accra (capital of Ghana) on the Gulf of Guinea (5°06′N 1°15′W; 5.1°N 1.25°W). The city has a population of 169,894 from 2010 Census (Ghana Statistical Service) and occupies approximately 122 sq km of land. The major rainy season is between May and July with mean monthly relative humidity varying between 85 and 99 %. In 2014, the city recorded about 74 days with rain, totalling about 963.8 mm (Regional Meteorological Office, Cape Coast, Ghana). Mean monthly temperature, relative humidity and total monthly rainfall during the study period is presented in Fig. 1.

### Larval survey and water quality assessment

**Fig. 2:**
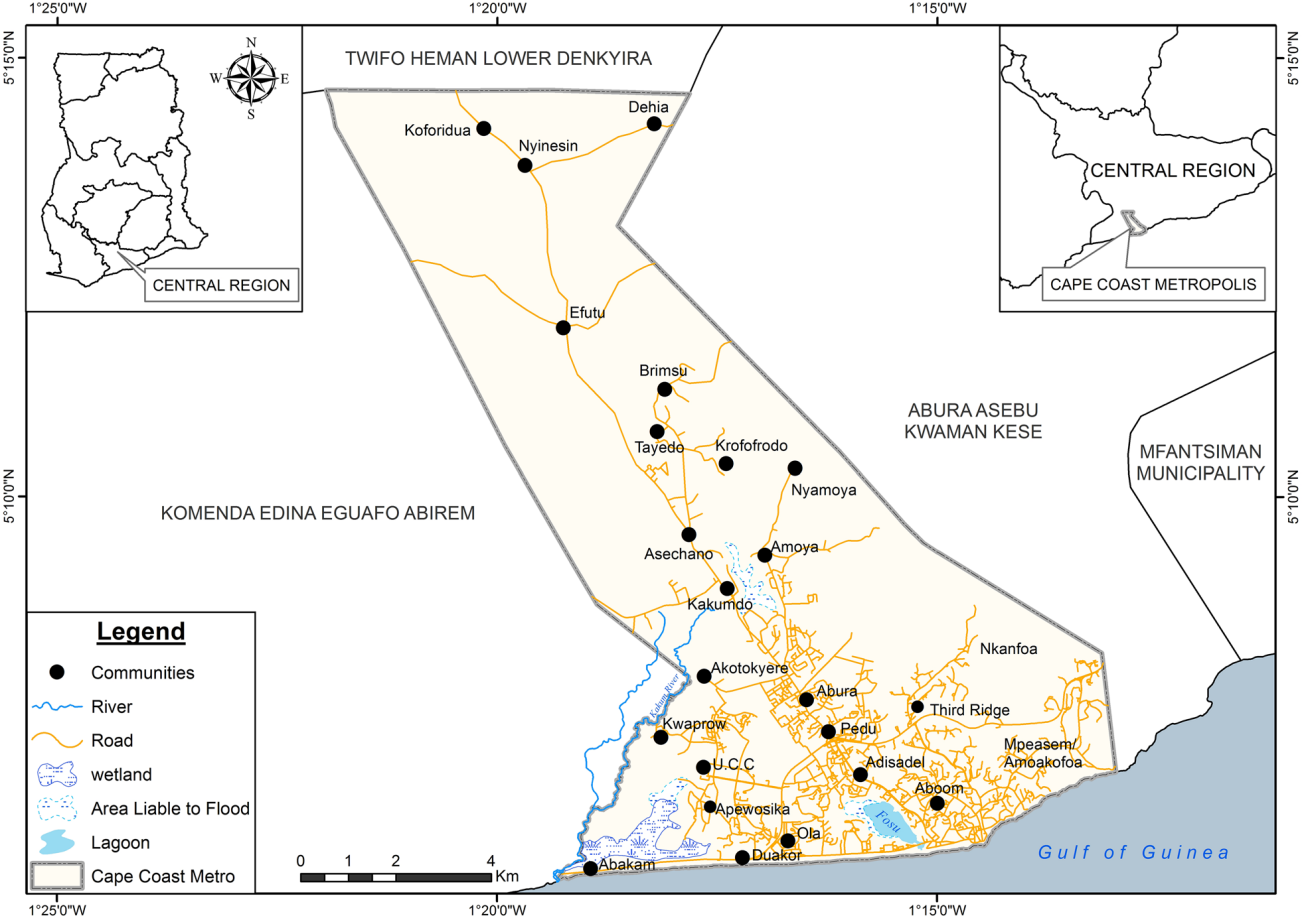
Map of Cape Coast Metropolis. Study sites were selected according to landscape (high/low altitude areas), land cover (swampy/non swampy areas) and land use settings (plan/unplanned residential areas and commercial areas). The study sites were: Third Ridge (high altitude area); UCC campus and Ola/Adisadel (residential areas); Duakor, Apewosika/Amamoma (shanty communities); Aboom/Kotokuraba (commercial areas); Nkafoa (peri-urban area); Abakam and Bakano (swampy areas)

A larval survey was conducted from January 2014 to March 2015. In the city, study sites were selected according to landscape (high/low altitude areas), land cover (swampy/non swampy areas) and land use settings (plan/unplanned residential areas and commercial areas) (Fig. 2). The larval survey was conducted to determine temporal and spatial distribution of *Anopheles* species in the study sites. There are two seasons in Ghana: dry and rainy, and a larval survey was conducted during the peak of each of the season. Dry season was a season of interest due to limited traditional habitats (water pools) for *Anopheles* species; the survey was conducted in two dry seasons: January–March 2014 and January–March 2015, and data for the rainy season were collected during May–July 2014.

Each study site was monitored once every fortnight for the three-month period for each of the three seasons (2014 dry season, rainy season and 2015 dry season). All open water bodies in the study sites were explored on both public and private properties. When a larval positive breeding habitat was found, the habitat was monitored once every fortnight for 2 months (if a larval positive habitat was found close to the end of a season, the two-month monitoring continued after the end of that season). For every visit, mosquito larvae were sampled and water quality characterized according to some selected water quality parameters. This monitoring protocol was done to gain insight into temporal dynamics of breeding habitats and the impact on species composition and spatial distribution of *Anopheles*, as well as to determine longevity of the breeding habitats.

Habitats were characterized using a standard format for each site, including water quality, nature and features of habitats, and presence or absence of vegetation in the breeding sites. Water quality was characterized by temperature, pH, dissolved oxygen, salinity, conductivity, and ammonia (non-ionized (NH3) and ionized (NH4^+^)) and bacteria fauna. These water quality parameters were selected to give basic physical characteristics of the breeding habitats as well as the level of organic pollution in the habitats. With the exception of total coliform, water quality parameters measured were performed in the field using a portable meter (YSI Professional Plus, YSI Inc, Yellow Springs, OH, USA and Oakton PCD650, Oakton Instruments, IL, USA). Using pour plate method, the concentration of bacterial population was determined in individual breeding habitats. Organisms showing similar morphological characteristics were sub-cultured. The Gram staining procedure was performed on all the individual and different (morphologically) colonies on Petri dishes to ascertain the cellular shape and arrangement. Individual colonies were identified according to the procedure described by Leboffe and Pierce [12].

Mosquito larvae collected from each breeding habitat were reared to adult in a separate and labelled rearing tray covered with netting and placed in an emergence chamber. The emergence chamber was made up of a wooden box covered with a 1-sq mm nylon mesh and a transparent roof. Light intensity, temperature and relative humidity in the chamber were similar to that of the outside environment. The larvae were reared in water taken from the habitat from which the mosquito was collected. GPS location were recorded with Garmin (eTrex^®^ 10) and iPad^®^ (Apple Inc.) using an application by Petosoft^®^.

### Assessing basic knowledge on the aquatic stages of mosquitoes and their breeding habitats

A qualitative study in the form of a short interview with property owners, where the larval survey was conducted on their properties, was conducted to assess their basic knowledge of the aquatic stages of mosquitoes and their breeding habitats. Most of the private properties comprised construction sites, uncompleted buildings and residential buildings without fencing. Facilities such as gutters, cesspits, open water-tanks/reservoirs, open wells, and newly constructed septic tanks which were yet to be covered, were explored for larvae. An interview lasting between 10 and 15 min was conducted for each of the participants. Ten participants were selected based on following criteria: (1) being present on their property during the larval survey; (2) accepted to be interviewed; and, (3) presence of mosquito larval habitat on their property. As part of the interview, the participants were: (1) shown mosquito larvae to identify; (2) asked to mention mosquito breeding places they knew of; and, (3) to describe the stages of mosquito development. Permits and approvals were not required for this study. However, permission was sought from private property owners or caretakers to conduct the larval survey on their properties.

### Molecular species identification

Adult mosquitoes that emerged from the larvae collected from breeding habitats were morphologically identified according to Gillies and Coetzee [13]. Further molecular identification was conducted on about 20 individuals from randomly selected habitats from each of the study sites. The PCR assay was conducted according to the procedure described by Scott et al. [14]. The two species, *An. coluzzii* (formerly *An. gambiae* M molecular form and hereafter *An. coluzzii*) and *An. gambiae* (formerly *An. gambiae* S molecular form and hereafter *An. gambiae*) were further identified using the SINE PCR method described by Santolamazza et al. [15]. *Culex* species were also morphologically identified and followed by PCR to confirm *Culex quinquefasciatus* using the method described by Smith and Fonseca [16]. Genomic DNA was extracted using QIAGEN DNeasy kit according to the manufacturer’s instructions.

### Data analysis

Physicochemical parameters and concentration of bacterial population were compared between *Anopheles*-positive breeding sites and habitats colonized by only *Culex* species using Mann–Whitney U test. The habitats were map using ESRI’s ArcMap software.

## Results

### Distribution of *Anopheles* species in Cape Coast

All the *Anopheles* larvae that were collected and reared to adult were morphologically identified as *An. gambiaes.l.* Out of 262 individuals that were identified by PCR assay, 91 % were *An. coluzzii* whereas 8.7 % were *An. gambiae*. *Anopheles melas* was about 0.3 % and was only found during the dry season (Table 1). Surprisingly, none of the 150 individual *Culex* species was positive for *C. quinquefasciatus* from the PCR diagnostic assay.

**Table 1:** *Anopheles* species composition over space and time in Cape Coast, Ghana

Habitat	Community	Date	Season	*Anopheles* species (N)
Pool of water	Bakano	January 2014		*An. coluzzii* (5)
Pond	Abakam	February 2014		*An. melas* (1)
Pool of water	Duakro	February 2014		*An. coluzzii* (20)
Stream	UCC campus	February 2014		*An. coluzzii* (20)
Pool of water	Apewosika	March 2014	Dry	*An. coluzzii* (4); *An. melas* (1)
Pool of water	Amamoma	March 2014		*An. coluzzii* (16)
Pond	Amamoma	March 2014		*An. coluzzii* (20)
Pool of water	Apewosika	March 2014		*An. coluzzii* (1)
Pool of water	Apewosika	May 2014		*An. coluzzii* (20)
Tyre track	Amamoma	May 2014		*An. coluzzii* (2); *An. gambiae* (3)
Rain pool	Adisadel	June 2014	Rainy	*An. coluzzii* (20)
Pothole	North Ola	June 2014		*An. coluzzii* (20)
Tyre track	North Ola	June 2014		*An. coluzzii* (20)
Pool of water	Abura	July 2014		*An. coluzzii* (20)
Choked gutter	North Ola	January 2015		*An. coluzzii* (20)
Stream	UCC campus	January 2015		*An. coluzzii* (9)
Concrete tank	North Ola	January 2015	Dry	*An. coluzzii* (20)
Gutter	Nkafoa	February 2015		*An. gambiae* (20)

### Water quality parameters in *Anopheles* breeding habitats

The median and interquartile range (IQR) of different water quality parameters measured in *Anopheles*-positive habitat (APL habitat) and habitats colonized by only *Culex* larvae are presented in Table 2. A two-tailed Mann–Whitney U test showed that APL habitats had higher temperature, pH, dissolved oxygen and lower ionized ammonia (NH4^+^) compared to breeding habitats with only *Culex* larvae. Furthermore, APL breeding habitats were slightly high in salinity with a median (IQR) of 0.8 ppt (0.4–2.3) (N = 72). Interestingly, about 48 % (22/46) *Culex* only habitats had no measurable dissolved oxygen (anoxic habitats) whereas APL breeding sites had a median (IQR) of 7.6 mg/l (4.6–8.8) of dissolved oxygen. Non-ionized ammonia (NH3) level was similar in the two habitats but ionized ammonia (NH4^+^) was significantly higher (p = 0.001) in habitats colonized by only *Culex* larvae compared to APL habitats (Table 2). The median (IQR) of bacterial concentration in APL habitats was 49 (36.5–59) × 10^3^ cfu/ml (N = 21), which was similar in habitats colonized by only *Culex* larvae, 42 (22–78) × 103 cfu/ml (N = 27) (p = 0.908). Most of the bacterial fauna that were identified belong to the thermotolerant (faecal) coliforms. Five species: *Escherichia coli, Clostridium* species, *Klebsiella pneumoniae, Enterobacter aerogenes*, and *Salmonella* species were present in all the individual habitats (Table 3).

**Table 2:** The median (IQR) of physicochemical parameters in mosquito breeding habitats in Cape Coast

Water quality parameter	Type of breeding habitats*			
	Pool of water (APL habitat) (N = 20)	Choked gutters (APL habitat) (N = 5)	All APL habitats^#^ (N = 72)	*Culex* only habitats^#^ (N = 46)
Temp °C	36.7 (35.6–37.9)	33.9 (33.3–34.4)	34.9^a^ (33.4–37.1)	31^b^ (29.5–32.4)
pH	7.6 (7.1–8.0)	8.8 (8.2–8.9)	8.1^a^ (7.3–8.6)	7.3^b^ (6.9–7.7)
DO mg/l	5.2 (3.1–6.8)	8.8 (8.5–11.5)	7.6^a^ (4.6–8.8)	0.2^a^ (0–0.8)
Sal ppt	0.87 (0.5–1.4)	0.94 (0.9–1.5)	0.8^a^ (0.4–2.3)	0.6^a^ (0.5–0.7)
SC µs/cm	1743 (977–2680)	1518 (1210–2668)	1452.5^a^ (793–4209)	1052^a^ (667–1439)
TDS mg/l	1134 (511–1742)	988 (953–1492)	952.9^a^ (435–2371)	600^a^ (521–979)
Res ohm–cm	466 (375–1055)	537 (394–546)	545.8^a^ (226–1169)	729.2^a^ (537–1010)
NH4^+^ mg/l^§^	3.4 (3–3.7)	16.5 (9.7–23.2)	3.3^a^ (2.4–4.2)	35.7^b^ (29.9–68.1)
NH3 mg/l^§^	0.5 (0.4–1.2)	3.2 (2.6–3.8)	0.6^a^ (0.2–1.8)	0.9^a^ (0.7–1.8)

* APL habitats (*Anopheles* positive habitats) comprises habitats that were either being colonized by only *Anopheles* or both *Anopheles* and *Culex* species, whereas *Culex* only habitats were habitats that were colonized by only *Culex* species

^#^ Values in ‘All APL habitats’ and *Culex* only habitats for each water quality parameter sharing same letter are not significantly different; (*IQR* inter-quartile range, *Temp* temperature, *DO* dissolved oxygen, *Sal* salinity, *SC* specific conductivity, *TDS* total dissolved solids, *Res* resistivity, *NH4* ^*+*^ ionized ammonia, *NH3* non-ionized ammonia)

^§^ Ammonia ions were not measured in habitats that had salinity of 1 ppt or more

**Table 3:** The median (IQR) of concentration of bacterial fauna in mosquito breeding habitats in Cape Coast

Type of breeding habitat^a^	Median (IQR) of total viable count (×10^3^ cfu/ml)	Bacterial species^b^
*Anopheles*-positive breeding habitats (N = 21)	49 (36.5–59)	*Enterobacter cloacae, Proteus mirabilis, Streptococcus faecalis, Shigella* sp*., Klebsiella oxytoca, Staphylococcus aureus, Escherichia coli, Clostridium* sp*., Klebsiella pneumoniae, Enterobacter aerogenes, Salmonella* sp.
*Culex* only habitats (N = 27)	42 (22–8)	*Citrobacter* spp*., Shigella* spp, *Streptococcus faecalis, Proteus mirabilis, Klebsiella oxytoca, Staphylococcus aureus, Escherichia coli, Clostridium* sp*., Klebsiella pneumoniae, Enterobacter aerogenes, Salmonella* sp.

^a^Bacteria fauna was assessed in these breeding habitats during the dry season (January–March 2014)

^b^Five species: *Escherichia coli, Clostridium* species, *Klebsiella pneumoniae, Enterobacter aerogenes*, *Salmonella* species were present in all individual habitats

**Fig. 3:**
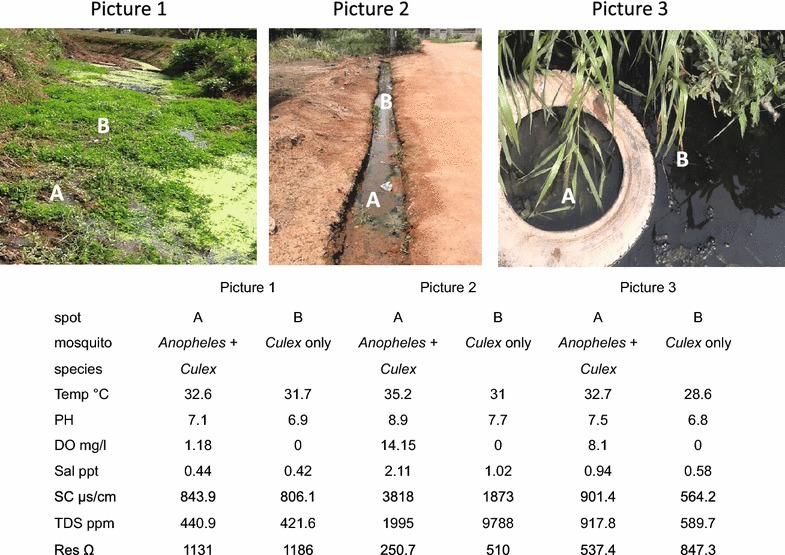
Spatial variation in water quality in mosquito breeding habitat in Cape Coast and its effect on distribution of *Anopheles* larvae in space over the breeding habitat. *Picture 1*—a stream on University campus, *Picture 2*—a gutter in Amamoma, *Picture 3*—a choked gutter in North Ola; *A* spot where *Anopheles* larvae were found, *B* spot where only *Culex* larvae were found

**Fig. 4:**
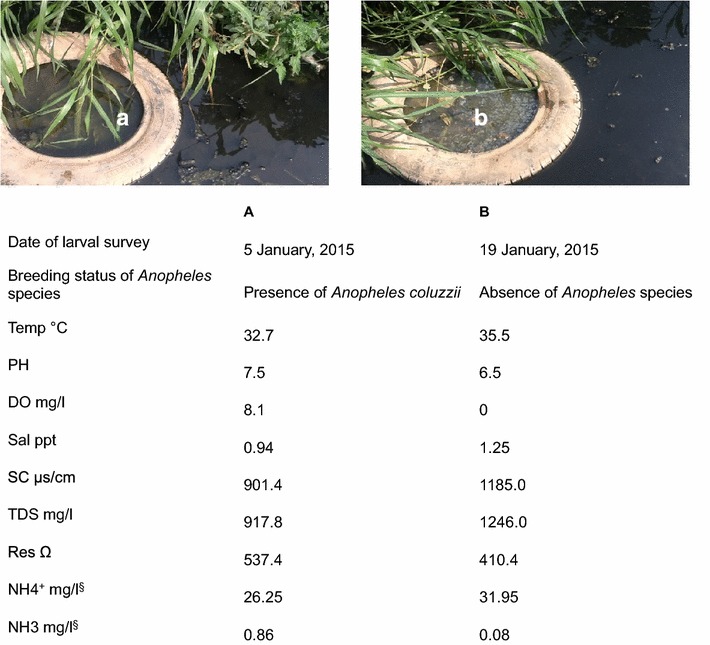
Temporal changes in water quality in a mosquito breeding habitat in Cape Coast and its effect on the distribution of *Anopheles* larvae in time in the breeding habitat. **a** water quality measured in 5 January, 2015; **b** water quality measure in 19 January, 2015

Within large APL breeding habitats, it was observed that *Anopheles* larvae were mostly confined to specific areas or spots whereas *Culex* larvae were relatively evenly distributed. Physicochemical measurements in the *Anopheles*-confined areas and other areas with only *Culex* larvae revealed variations in water quality (Figs. 3, 4). This was more evident in the level of dissolved oxygen and temperature. Dissolved oxygen and temperature were higher in specific areas where *Anopheles* larvae were mostly found compared to areas with only *Culex* larvae.

### Nature of *Anopheles* breeding habitats

**Fig. 5:**
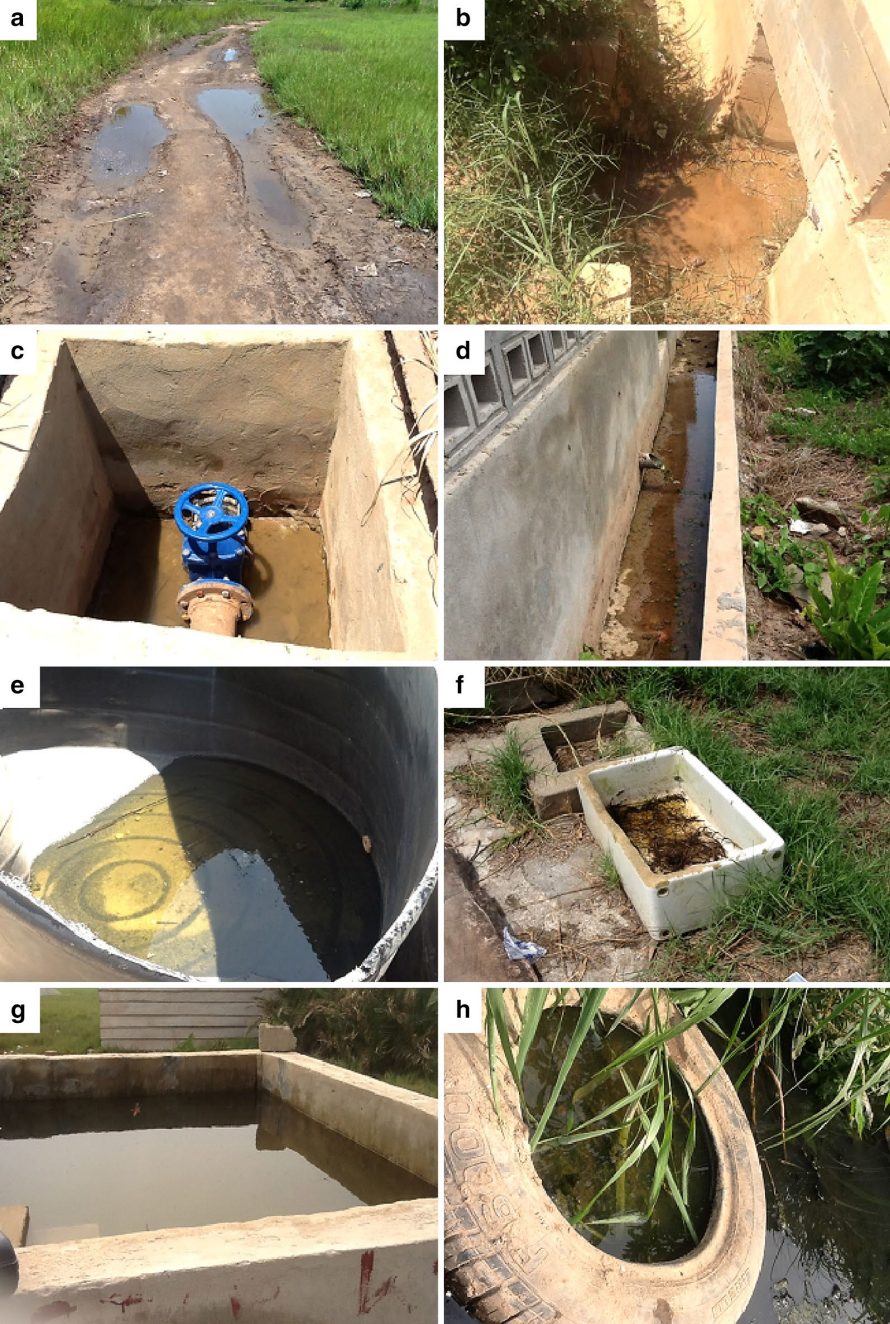
Some major *Anopheles* positive breeding habitats in Cape Coast. During the rainy (**a**–**d**) and dry (**e**–**h**) seasons; **a**, **b** water puddles on a public road and waterway; **c** water pool in a concrete receptacle holding a stopcock of a public water pipeline; **d**: poorly drained gutter in a private residential property; **e**, **f** abandoned water-holding containers on private properties; **g** concrete water tank at a private construction site; **h** choked gutter with high input of solid and liquid waste from households

Several water-holding facilities in houses and at construction sites, wells, broken pipes, choked gutters, and a wide range of water pools were the major *Anopheles* larval habitats in the study sites (Fig. 5).

Large numbers of APL habitats in the form of puddles, tyre tracks, rain pools, and potholes were observed. It appears most depressions that can hold water during the rainy season were colonized by *Anopheles*. For example, within a 100-m section of a road (Fig. 5a), about 50 individual APL habitats were counted and several of such roads were observed in the study sites. Interestingly, most of these habitats were on public properties, such as public roads and constructed waterways (Fig. 5b).

During the dry season, APL habitats were mostly choked gutters and different types of water receptacles, including concrete and plastics containers. Some transitory habitats were also found after a few rainfalls recorded in the two dry seasons. The first dry season (January–March 2014) recorded about 13 days of rainfall totalling 127.2 mm, whereas the second dry season (January–March 2015) recorded about 11 days of rainfall totalling 154.5 mm. The rainfall pattern differs slightly between the two dry seasons, which consequently affected the distribution of APL habitats in the two seasons. For example, only a single rainfall of 5 mm was recorded for January 2015 whereas 3 days of rainfall of 36.8 mm was recorded in January 2014. Hence, it was not surprising that transitory habitats such as water puddles and water pools were recorded in January 2014 but only gutters and water reservoirs (Fig. 5g) were recorded in January 2015.

**Fig. 6:**
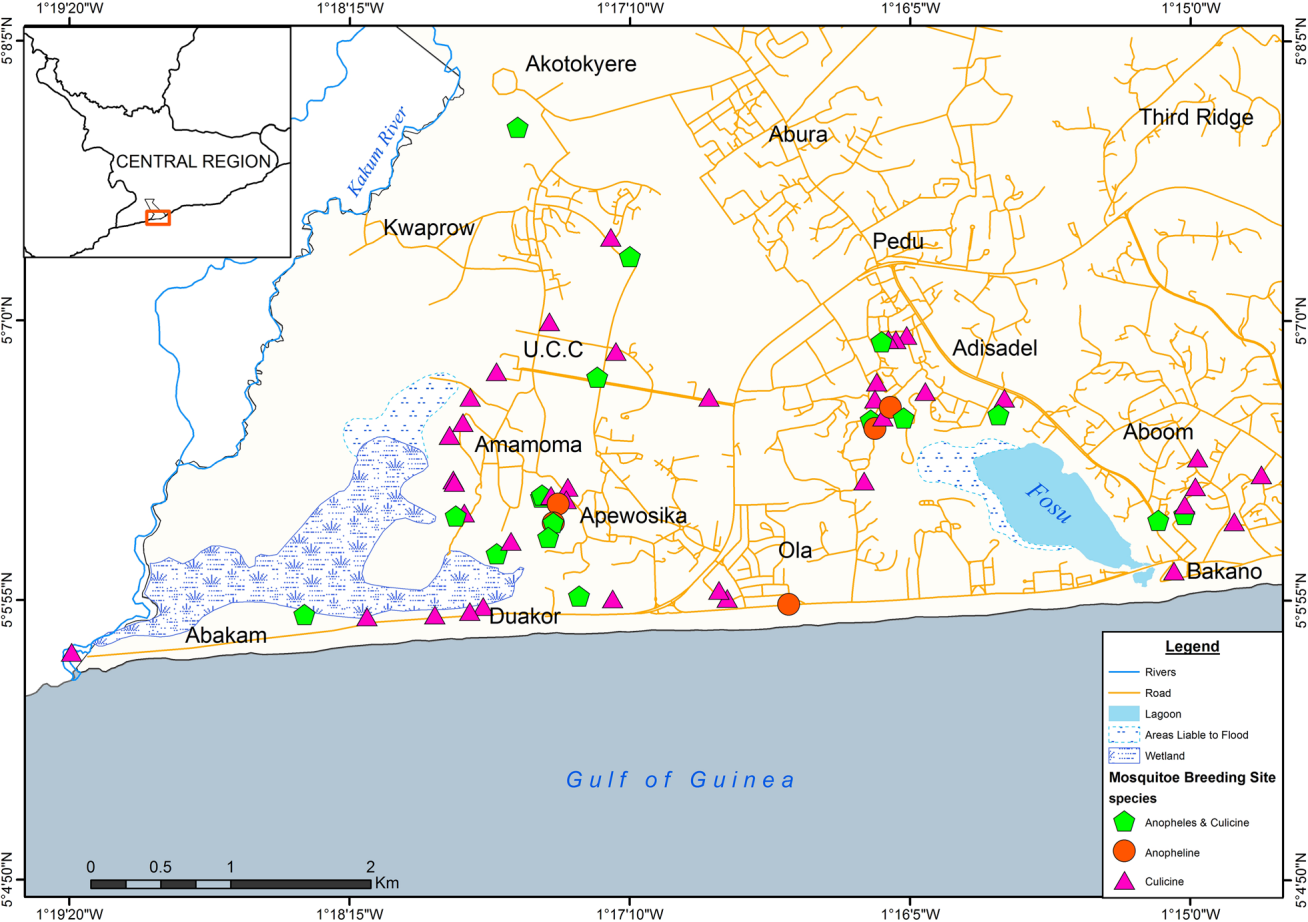
Distribution of mosquito breeding habitats in study communities in Cape Coast. Spatial distribution of mosquito breeding habitats in Cape Coast during the dry season in study communities (*Anopheles*-positive habitat = anopheline + *Anopheles* and culicine)

**Fig. 7:**
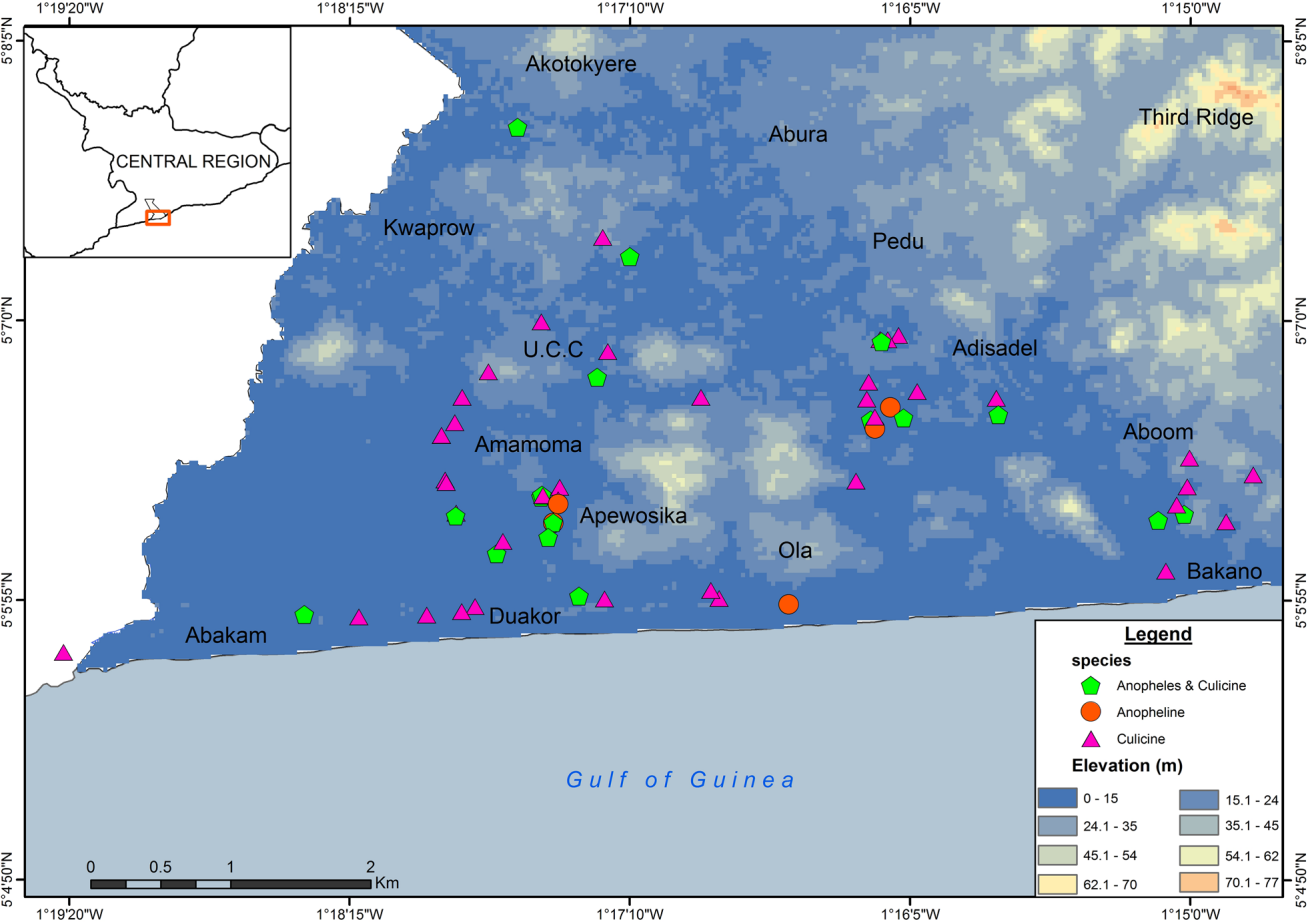
Distribution of mosquito breeding habitats according to elevation in Cape Coast. Spatial distribution of mosquito breeding habitats in Cape Coast during the dry season according to elevation (*Anopheles*-positive habitat = anopheline + *Anopheles* and culicine)

The spatial distribution of mosquito breeding habitats during the dry season is presented in Fig. 6. The mean ± SD elevation where APL habitats were found was 11.5 ± 2.3 m (Fig. 7). Most APL habitats were found around lowland areas or areas close to wetlands.

The longevity of habitats varied greatly. Some breeding habitats existed for a few days, some weeks and others throughout the year. While some habitats were colonized by *Culex* larvae throughout the study period, none of the breeding habitats was found harbouring *Anopheles* larvae for an entire season. Besides habitats drying up, the conditions of some of the habitats changed and this in turn affects the colonization of *Anopheles* larvae in time. The two main factors that were observed to cause changes in habitat conditions were changes in water quality (Fig. 4) and vegetation cover (weeds covering the habitat). Also, heavy downpour was a major factor during the rainy season.

### Basic knowledge on the aquatic stages of mosquitoes and their breeding habitats

None of the ten private property owners/caretakers was able to correctly identify the mosquito larvae shown to them nor able to describe the developmental stages of a mosquito. Most of them have seen mosquito larvae before but wrongly thought they were tadpoles (immature stage of a frog), worms or something other than a mosquito. However, they all know that mosquitoes breed in stagnant water bodies but were not aware that concrete tanks and other water-holding receptacles on their properties were breeding mosquitoes. For instance, when asked to describe the developmental stages of a mosquito, one participant who was a nurse by profession said

*“I know they lay eggs in stagnant waters. I am not sure what happens after that, but, I think immediately after the eggs hatches they fly away*”. Like others, she only knew the adult stage of a mosquito.

## Discussion

Understanding the ecological drivers that are responsible for modulating the distribution and habitat segregation of *Anopheles* species is of paramount importance in malaria control. In the present study, *An. coluzzii, An. gambiae* and *An. melas* belonging to *An. gambiae* complex were found in Cape Coast but their abundance and distribution varied. *Anopheles coluzzii* dominated in almost all the breeding habitats and was found actively breeding in both rainy and dry seasons in diverse habitats. Habitats that were colonized by *An. coluzzii* during the rainy season (footprints, tyre tracks, rain pools) are consistent with the known preference of *An. gambiae*, particularly in rural areas [17]. However, salinity was relatively high in breeding habitats found in this study. In the dry season, *An. coluzzii* was mostly found cohabiting with *Culex* species in choke gutters and other organic polluted habitats, which was indicated by high levels of ammonia ions. This is not surprising because from both laboratory experiments [18] and field observations [6, 10, 11, 19], it is becoming clearer that *An. coluzzii* exhibit some degree of tolerance to high salinity and ammonia ions.

The high prevalence of *An. coluzzii* in this study is consistent with previous studies in the country. Previous studies in Ghana have shown *An. gambiae* and *An. coluzzii* sympatrically co-existing in most locations. However, *An. gambiae* predominate in the central part of the country whereas *An. coluzzii* and *Anopheles arabiensis* predominate in the northern and coastal savannah regions, with *An. melas* occurring mainly at the coastal areas [20, 21]. The result also conforms to the continental species distribution model that has been described by Tene-Fossog and colleagues [22], which predicted predominance of *An. coluzzii* along the coastline of west and central Africa. High level of salinity observed in the breeding habitats colonized by *An. coluzzii* may have contributed to its high prevalence in Cape Coast [22]. The only habitat that *An. gambiae* dominated was found at the peripheral part of the city, away from the core of the densely urbanized area and away from the coast.

The dominance of *An. coluzzii* in coastal areas of Ghana has been explained by the wide presence of permanent breeding conditions from irrigation facilities and ponds, resulting from river run-off [20, 21]. Similarly, along its geographical distribution, *An. coluzzii* is seen to predominate in areas characterized by larger, more temporally stable breeding sites, such as rice paddies and irrigation facilities [23–25]. On the contrary, this study found *An. coluzzii* breeding in small ephemeral habitats similar to that described for *An. gambiae*. Additionally, *An. coluzzii* and *Culex* larvae co-existed in marginal habitats such as organic polluted habitats, particularly during the dry season. The absence of *An. coluzzii* in marginal habitats during the rainy season may suggest that it also has preference for temporary aquatic habitats. However, in the absence of preferred habitat, its ability to tolerate high ions such as ammonia and salinity, perhaps, makes it possible to breed in marginal habitats to maintain its population during unfavourable season.

Considering the diverse nature of APL habitats and different levels of water quality in breeding habitats, further insight could be gained from investigations of chronic exposure of *An. coluzzii* to organic pollution and its impact on life–history traits. Mireji and colleagues [26] showed that *An. gambiae* that developed tolerance to some heavy metals occurred at a significant biological cost to the mosquito, adversely affecting its ecological fitness. Similarly, intensive mortality was observed in the early instars of *Culex tarsalis* that bred in poor water quality habitats [27]. Surprisingly, *C. quinquefasciatus*, a common mosquito species in organic polluted waters, was absent in this study and it is consistent with a previous study in Cape Coast [28]. The reason for the absence of this cosmopolitan and widely distributed species is not presently clear.

Water quality in the breeding habitats seems to be affected by natural and human influences. On the one hand, the habitats were high in salinity, electrical conductivity and total dissolved solids, which may have been as a result of proximity to the sea, although anthropogenic factors cannot be ruled out. On the another hand, low dissolved oxygen, high ammonia ions and high concentration of thermotolerant bacterial coliforms in some APL habitats may have been caused by organic pollution resulting from high input of solid and liquid waste from households. Presence of faecal bacteria such as *Escherichia coli*, faecal streptococci and other coliforms also confirmed faecal contamination in the breeding habitats. Nevertheless, a lower level of total ammonia in APL habitats compared to habitats colonized by *Culex* larvae only and the absence of *An. coluzzii* in anoxic breeding habitats suggests that its tolerance to organic pollution is probably lower than *Culex* larvae. This is further supported by the distribution of *An. coluzzii* in space and time in marginal habitats, which appears to be influenced by some level of water quality, notably temperature and dissolved oxygen (Figs. 3, 4). Interestingly, similar levels of non-ionized ammonia (NH3) were found in APL habitats and habitats colonized only by *Culex* larvae, which could indicate that the toxic effect of ammonia might be similar in both species since non-ionized ammonia is the principal toxic form of ammonia to organisms.

The rapid urbanization being witnessed in many cities in Africa is associated with pollution and environmental modification. This can create or modify various mosquito larval habitats, which in turn can affect vector composition or distribution. Unlike Cameroon where the impact of urbanization on the prevalence of *An. coluzzii* has been extensively studied [11], there is no such detailed information available in Ghana and most West African countries. The two highly urbanized areas in the country, Accra (coastal savannah zone) and Kumasi (forest zone) are dominated by *An. coluzzii* and *An. gambiae*, respectively. From previous studies, ecological influence rather than urbanization appears to be of greater importance in the distribution of *An. gambiae* species in Ghana [20, 21, 29]. Ecological factors such as elevation, precipitation and temperature have been shown to be important variables driving the spatial distribution of each of the *An. gambiae* species [22]. A country-wide assessment of water quality and species distribution may give a clearer picture of the role of urbanization in modulating the distribution of *An. gambiae* species in Ghana. Presently, there is limited information of water quality levels in *Anopheles* breeding habitats in the country. For example, data on level of salinity, ammonia ions and other water quality indicators in *Anopheles* breeding habitats are virtually absent for the central to northern part of Ghana. The few existing data are mostly found for the coastal areas [30–32].

Most of the APL habitats encountered in this study are consistent with reports from larval surveys conducted piecemeal in other urban areas in Ghana [6, 8, 30, 32] and several urban African countries [10, 11, 33]. Although habitat productivity, which was not assessed in this study, could differ from one habitat to other, it was more evident that the importance of habitats shifted from one habitat type to other with time since no habitat was continuously colonized for an entire season. It seems each type of breeding habitat, regardless of its level of productivity or importance, contributed to the production and maintenance of *Anopheles* species throughout the year. Most of the breeding habitats found in this study occurred as a result of haphazard and uncontrolled human activities. With the nature of breeding habitats in the study area, larval control intervention could greatly reduce *Anopheles* population in the city, particularly during the dry season. This may require constant monitoring of larval habitats and improvement of sanitation conditions in the city with regards to waste management. Ironically, there are in existence sanitation and by-laws in Ghana (Ghana Public health Act 851, 2012) that could prevent the formation of many of the *Anopheles* breeding habitats, however they are rarely enforced. Moreover, from the qualitative study with some of the private property owners, it appears they have inadequate knowledge of the immature stages of mosquitoes and their breeding habitats. Yet, community engagement is one of the most important aspects of mosquito management programmes. Therefore, the following: (1) proper waste management and maintenance of public roads; (2) strict enforcement of hygiene and sanitation by-laws; and, (3) community education on mosquito biology and their breeding places in the local environment could prevent formation of many mosquito breeding habitats, which could make larval control more feasible and cost effective in Cape Coast.

## Conclusions

*Anopheles coluzzii* was found breeding in organic or faecal polluted habitats. Water in most of the breeding habitats either had high level of salinity or ammonia ions, which may be related to the proximity to the coast or urbanization, respectively. Furthermore, high concentration of bacterial fauna including several species of faecal coliforms confirms presence of organic pollution and faecal contamination in *An. coluzzii* breeding habitats. Organic pollution was lower in *An. coluzzii* breeding habitats than *Culex* only habitats. The nature of breeding habitats found in the city demonstrates the opportunistic behaviour of *An. coluzzii* and how its breeding requirements are so intimately intertwined with the haphazard and uncontrolled human activities in the urban area. Considering the nature of APL habitats, improving basic hygiene and sanitation in the city could make larval control intervention more practical and cost effective.
